# Psychological Distress among Pharmacists during Second Wave of Pandemic: A Cross-Sectional Study

**DOI:** 10.1155/2022/3606351

**Published:** 2022-12-27

**Authors:** Keshav Dhakal, Shobhana Nepal, Pratigya Sapkota

**Affiliations:** ^1^Department of Pharmacy and Pharmacology, Chitwan Medical College Teaching Hospital, Bharatpur, Nepal; ^2^Shree Medical and Technical College, Bharatpur, Nepal; ^3^Bharatpur Central Hospital Pvt. Ltd, Bharatpur, Nepal

## Abstract

**Background:**

The outbreak of COVID-19 and subsequent lockdown worldwide have shown a psychological impact among healthcare workers. However, data on the psychological impact among community pharmacists are lacking in the Nepalese context.

**Aim:**

This study aimed to assess the psychological distress among community pharmacists during COVID-19 pandemic. *Settings and Design*. A cross-sectional analytical design was adopted to study the psychological distress of pharmacists working in community pharmacies in Bharatpur.

**Materials and Methods:**

Purposive sampling method was used to select 172 community pharmacists. The COVID-19 peritraumatic distress index (CPDI) questionnaire adapted from the Shanghai Mental Health Centre was used to measure psychological distress. *Statistical Analysis*. Data were analyzed using Statistical Package for Social Sciences (SPSS) version 20. Descriptive statistics (mean, standard deviation, percentage) and inferential statistics (chi-square and logistic regression) were used.

**Results:**

Out of 172 respondents, 77.9% were severely distressed while 22.1% were mild to moderately distressed due to COVID-19 pandemic. Mean age of the respondents was 27.81 ± 6.35 years. Logistic regression revealed that having education of bachelor and above (AOR = 4.489, 95% CI: 1.747, 11.539), 8 or more working hours (AOR = 7.633, 95% CI: 2.729, 21.352), being unsatisfied with the job (AOR = 11.524, 95% CI: 3.574, 37.158), and having experience of more than 3 years (AOR = 2.857, 95% CI: 1.060, 7.702) were found significantly linked to severe psychological distress among community pharmacists in Bharatpur.

**Conclusion:**

All respondents had suffered from some degree of psychological distress due to the pandemic. Our findings reveal the need for psychological intervention to alleviate psychological distress among pharmacists.

## 1. Introduction

Coronavirus disease (COVID-19) has taken a heavy toll on people's health and lives, affecting severely healthcare systems all across the globe. COVID-19 not only threatened people's health but also gave rise to various psychological complications such as anxiety, depression, and panic disorder [1–4].

During the tough period of COVID-19, pharmacy professionals were well acknowledged by the public and healthcare system as an essential professional of the frontline healthcare system [4, 5].

Being one of the frontline healthcare workers, pharmacists untiringly delivered much-needed health services during the period of pandemic. Community pharmacists are responsible for COVID-19 screening and medication dispensing, disseminating important information about COVID-19, working in close coordination with other healthcare workers and government organizations, home delivery of medications whilst remaining the most approachable healthcare member that patients can contact during the pandemic [5–8].

A study conducted in Spain revealed a high prevalence (80.6%) of psychological distress among healthcare professionals during the COVID-19 health crisis [9]. A study conducted among 681 healthcare workers in Italy found that 49.38% had post-traumatic stress symptoms; 24.73% had symptoms of depression; 19.80% had symptoms of anxiety; 8.27 had insomnia; 21.90% had high perceived stress [10]. Even the study findings from Jordan, a country with low caseload, have revealed that 32% healthcare workers experienced high distress and 20% experienced severe distress. Exhaustion, anxiety, depression, and sleep disturbances were reported by approximately 34%, 34%, 19%, and 29% of subjects, respectively [11].

Not only the immediate effects but also few past studies have elucidated about the long-term effects of previous virus epidemics. These studies revealed that the long-term sequel of SARS outbreak of 2003 occurred among high-risk healthcare workers with them remaining highly stressed, and this high stress level was associated with higher levels of depression, anxiety, and posttraumatic scores after 1–3 years [12–14].

Previous studies have shown a high level of psychological distress in healthcare professionals. However, data on mental health issues of pharmacists are lacking in Nepalese setting. Thus, this study was attempted to assess the level of psychological distress among pharmacists during a pandemic and its associated factors.

## 2. Methodology

### 2.1. Study Design, Setting, and Sampling Technique

A cross-sectional analytical study was conducted to assess psychological distress among community pharmacists during the second wave of COVID-19 pandemic in Bharatpur, Nepal. Data were collected from 11 August to 26 August 2021, which was during the second lockdown period in Nepal.

The setting for this study was Bharatpur Municipality, which is located in the western bank of Narayani River in Chitwan District of Bagmati province, Nepal. There are approximately 652 registered allopathic pharmacies in Bharatpur Municipality where ward number 10 has the maximum number of pharmacies, i.e., 207 followed by ward number 7 with 72 pharmacies. Therefore, we selected these two wards purposively for data collection. Data were collected from one pharmacist from each pharmacy selected. Pharmacists who had completed Diploma in Pharmacy, Bachelors in Pharmacy, and Masters in Pharmacy and registered in Nepal Pharmacy Council, belonging to the age group of 18–59 years and working in community pharmacies for more than one year were included in the study. Owners as well as employee pharmacists were involved. Researchers visited the community pharmacy and distributed the questionnaire to the pharmacists working there and asked them to fill it. Self-administered tool was used to collect the data. The COVID-19 Peritraumatic Distress Index (CPDI) questionnaire adapted from the Shanghai Mental Health Centre was used to measure psychological distress [15]. Written informed consent was taken from all respondents before data collection. Questionnaire was collected after two days by the researchers themselves. Data were collected only after the ethical approval from Shree Medical and Technical College-Institutional Review Committee (SMTC-IRC) (SMTC-IRC-20210623-75).

#### 2.1.1. Sample Size

Sample size was determined using the following formula:

(1) $$\begin{array}{c} N=z^{2}*P\frac{\left(1-P\right)}{e^{2}}\text{,} \end{array}$$

where *z* is 1.96 at 95% confidence interval, *e* is the margin of error at 5%, and *P* is the prevalence rate of 11.5% from a previous study done in Nepal [2]. Adding 10% as nonresponse rate, the desired sample size obtained is 172.

### 2.2. Study Variables

Sociodemographic variables such as age (less than 30, 30–45, and above 45 years), sex (female and male), religion (Hinduism, Buddhism, Islam, and Christianity), ethnicity (Dalit, Janjati, Madhesi, Muslim, Brahmin/Chhetri, and others), educational qualification (D. Pharmacy, B. Pharmacy, and M. Pharmacy), and marital status (unmarried, married, divorcee, widow/widower) were included. In addition, work-related variables such as experience (0–4, 5–10, >10 years), position (owner and employee), hours of work per day (less than 8 and 8 or more hours), days per week (5, 6, and 7), satisfaction with job (yes, no), increase in workload (yes, no), extra allowance during pandemic (yes, no), and presence of chronic illness (yes, no) were elicited from the respondents.

To assess the psychological distress of the pharmacists, CPDI was used which is a standard tool and already validated in our setting, and its internal reliability was found to be very high (0.896) in a previous study [2].

CPDI consists of 24 statements with a 5-point Likert scoring system with never: 0, occasionally: 1, sometimes: 2, often: 3, and always: 4. Scores of 0–28 indicate normal or no distress. Scores between 29 and 51 indicate mild-to-moderate distress, while a scores greater than or equal to 52 indicate severe distress.

### 2.3. Statistical Analysis

The collected data were coded, checked, reviewed, and organized daily for completeness. Incomplete questionnaires were taken to respondents to fill up again the next day. Coded data were entered in Microsoft Excel and exported to SPSS version 20. Data were analyzed by using descriptive statistics (frequency, percentage, mean, and standard deviation) and inferential statistics (chi-square test and logistic regression to test the association between variables).

Binary logistic regression was applied to determine the association of various factors with level of psychological distress due to pandemic. Variables with a *p* value <0.05 were further computed for multiple logistic regression. Finally, variables with *p* value <0.05 in multiple logistic regression were determined as being factors significantly associated with psychological distress.

## 3. Results

A total of 172 pharmacists working in community pharmacies were included in this study. The demographic and work-related data of the respondents are shown in Table 1. The majority (70.9%) of the respondents were less than 30 years old, and the mean age of the participant was 27.81 ± 6.35 with min age 19 and max age 54. The male to female ratio was 0.69 with 59.3% of female respondents. The majority (82.0%) of the respondents were Hindu by religion. Nearly half (48.3%) of the respondents were Brahmin/Chhetri. More than half (55.8%) of the respondents were unmarried while there were no widows and widowers. Similarly, more than half (51.7%) of the respondents had completed a diploma level in Pharmacy. Four-fifths (79.7%) of the respondents were employees at community pharmacies whereas the rest were the owners of it. Mean working hours per week was 59.15 hours. Respondents who worked for 6 days were 58.1%. Likewise, 72.1% of the respondents worked 8 or more hours a day, and 80.2% reported an increased workload. Despite this, 73.8% of the respondents reported not receiving any extra allowance during the pandemic. Respondents satisfied with their job were 64.0%. The majority (94.8%) of the respondents had no chronic illness.

Table 2 shows the distribution of respondents on COVID-19 Peritraumatic Distress Index. Majority of the respondents reported feeling more nervous and anxious (87.8%) and felt unsecured and bought a lot of masks, medications, sanitizers, gloves, and/or other home supplies (88.4%). More than two-thirds of them reported feeling helpless no matter what they did (70.3%) and avoided watching COVID-19 news since they were too scared to do so (70.3%). Majority (91.9%) felt sympathetic to COVID-19 patients and their families. Less than two-third believed COVID-19 information from all sources without any evaluation (62.2%) and felt uncomfortable when communicating with others (62.8%). Majority found it hard to concentrate (82%) and make decisions (82%). More than half reported feeling stomach pain, bloating, and other stomach discomforts often (57%) and talked with their family members very rarely (50.6%).

Table 3 depicts the distribution of pharmacists on the severity of psychological distress. All respondents had some degree of psychological distress. More than three-fourth (77.9%) respondents had severe distress followed by mild to moderate distress in 22.1%. There were no respondents who had no psychological distress.

Table 4 demonstrates the association between level of psychological distress and sociodemographic and work-related characteristics of pharmacists. There was significant association between level of psychological distress and variables such as age (*p* value 0.041), education (*p* value 0.007), working hours per day (*p* value 0.002), experience (*p* value 0.018), and satisfaction with job (*p* value 0.003). However, sociodemographic characteristics such as sex, ethnicity, religion, marital status, position, work days per week, workload, extra allowance, and chronic illness were not significantly associated with distress level.

Table 5 demonstrates logistic regression analysis showing that having an education of bachelor and above (AOR = 4.489, 95% CI: 1.747, 11.539), 8 or more working hours (AOR = 7.633, 95% CI: 2.729, 21.352), being unsatisfied with the job (AOR = 11.524, 95% CI: 3.574, 37.158), and having experience of more than 3 years (AOR = 2.857, 95% CI: 1.060, 7.702) were found significantly linked to severe psychological distress among community pharmacists in Bharatpur.

## 4. Discussion

In this study, 77.9% respondents had severe psychological distress followed by 22.1% with mild-to-moderate distress as measured by COVID-19 Peritraumatic Distress Index (CPDI). This finding is in line with the study conducted in Spain, which revealed severe psychological distress among 70% of community pharmacists [16]. Similar findings were reported in a study in South Africa where out of 953 pharmacists, 66.1%, 62.9%, 73.8%, and 51.3% had anxiety, depression, stress, and low quality-of-working life, respectively [17].

A study done to estimate and compare stress, anxiety, depression, and psychological impact before and after COVID-19 lockdown among frontline health workers in Hyderabad, India, revealed the highest level of PTSD and stress and the second highest level of depression among pharmacists during the lockdown period compared to other groups of health professionals with a considerable increase after the lockdown situation [18].

However, the findings of an online survey among 1006 healthcare workers including pharmacists (16.8%) in Jordan using Kessler-6 revealed 32% respondents suffered from high distress while only 20% suffered from severe distress [11]. Similar findings were reported in an online survey in China conducted among 4,219 hospital pharmacists where only 41.9% and 29.4% experienced mild to severe levels of anxiety and depression, respectively [19].

Similarly, a study conducted in Saudi Arabia among 501 health professionals including pharmacists (half of the study population) revealed the presence of depression, anxiety, and stress among 54.69%, 60.88%, and 41.92% of the respondents, respectively, which was conducted after one year of the pandemic [20].

Meanwhile, an online-based study on COVID-19 knowledge and pandemic-associated distress among 365 pharmacists in China using World Health Organization Self-Reporting Questionnaire revealed only 18.4% pharmacists met the Self-Reporting Questionnaire SRQ-20 threshold for distress [21].

However, the finding of this study is much higher than the nationwide web-based study conducted in Nepal among 254 health professionals using COVID-19 peritraumatic index (CPDI) including pharmacists which revealed 46.5% had mild-to-moderate distress while only 6.7% respondents had severe distress [3].

Similarly, another web-based survey in Nepal among 475 healthcare workers using HADS and insomnia severity index showed 41.9% had symptoms of anxiety, 37.5% had depression, and 33.9% had insomnia [22].

Studies from other countries have also shown a lower prevalence of psychological issues among frontline health professionals. A web-based survey among 1094 healthcare workers in Pakistan including pharmacists (6.9%) using patient health questionnaire (PHQ9) and generalized anxiety disorder-7 (GAD-7) revealed 45.4% had mild level of depression while 12% had a moderate-to-severe level. Moreover, 33.3% had moderate-to-severe levels of anxiety [23].

However, a study conducted in France among 135 community pharmacists using perceived stress scale, impact of event scale-revised, and Maslach Burnout Inventory revealed that only 35% pharmacists reported psychological problems. Seventeen percent reported significant posttraumatic stress. High burnout symptoms were found in 25%, 34.9%, and 3% respondents, respectively, according to emotions exhaustion, depersonalization, and personal accomplishment scores [24].

Individual studies done among other groups of health professionals such as nursing staffs and laboratory professionals have shown much lower prevalence of psychological distress in various countries including Nepal as compared to this study [25–29].

The prevalence of psychological distress among pharmacists was much higher in our study than most of the previous studies. The differences may be owing to the sample selection in previous studies where the health professionals included more number of physicians and nurses compared to pharmacists who were in minimal proportions. Thus, the psychological issues of pharmacists may have been under represented. In addition, the time of data collection plays a critical role. Various studies have shown an increased level of psychological distress among frontline health workers [30, 31] as well as general population after the first lockdown [32–34] and consequently more so during the second wave.

Since this study was conducted during the second lockdown period in Nepal, the cumulative effects of stress may have been reflected in the findings.

Furthermore, the use of different measuring tools to evaluate psychological distress may have influenced the variations.

In this study, education of bachelor and above was significantly associated with severe psychological distress. The reason may be those with higher educational qualifications might be having more responsibilities during their duties. This is in line with previous studies conducted among health workers including pharmacists [35, 36].

However, a previous study among community pharmacists and pharmacy technicians in Spain did not show association between education and level of psychological distress [16]. Similarly, few previous studies conducted in Nepal among health workers do not support this finding [2, 22, 37].

Having working hours of 8 or more was significantly associated with severe psychological distress. This is in line with many previous studies which have shown that long working hours negatively affect our mental health [38, 39]. The reason for this may be that as the working hours increase, job requirements and eventually chances of exposure to COVID increase considerably. However, several other studies have not reported an association between long working hours and psychological distress during the pandemic [3, 22].

Likewise, not being satisfied with the work was significantly linked with severe psychological distress, and this is in line with previous studies of Obedait et al. [40] and Hawari [11]. Job satisfaction has been linked to positive mental health in many previous studies [41, 42].

Furthermore, having experience of more than 3 years was significantly associated with severe psychological distress. This is consistent with the findings of a previous study in Nepal [22]. However, few other previous studies did not show any association between work experience and psychological distress among health workers during pandemic [17, 43].

The findings from this study provide an insight into the influence of a pandemic on psychological health among pharmacists specifically and emphasize the necessity of appropriate intervention to prevent psychological problems among this group of frontline health professionals in Nepal.

### 4.1. Limitations of the Study

The current study has several limitations. First, this is a cross-sectional study so the temporal link between the various factors and psychological distress cannot be measured as they are both examined at the same time. Moreover, a history of mental illness was not elicited from the respondents.

Second, using a purposive sampling technique to select the study area may have led to selection bias and limited the generalizability of the findings.

Despite these limitations, to the best of researchers' knowledge, this is the first study to measure psychological distress only among pharmacists in a Nepalese setting.

## 5. Conclusion

Community pharmacists like other frontline health workers are severely affected by the pandemic. Psychological distress among pharmacists has been found to be very high. Furthermore, educational qualification, working hours per day, years of experience, and satisfaction have been significantly associated with the level of psychological distress. The findings of this study highlight the need for providing support services to pharmacists to lessen the gravity of the psychological impact of this pandemic.

## Figures and Tables

**Table 1:** Sociodemographic and work-related characteristics of pharmacists.

Characteristics	Category	Frequency (%)
Age groups (in years)	Below 30	122 (70.9)
	30–45	45 (26.2)
	Above 45	5 (2.9)

Mean age ± S.D.	27.81 ± 6.35	
	Min: 19	
	Max: 54	

Sex	Male	70 (40.7)
	Female	102 (59.3)

Religion	Hinduism	141 (82.0)
	Buddhism	19 (11.0)
	Islam	6 (3.5)
	Christianity	6 (3.5)

Ethnicity	Dalit	19 (11)
	Janjati	46 (26.7)
	Madhesi	10 (5.8)
	Muslim	6 (3.5)
	Brahmin/Chhetri	83 (48.3)
	Others (giri, puri, sanyashi)	8 (4.7)

Education	Diploma in pharmacy	89 (51.7)
	B. Pharmacy	72 (41.9)
	M. Pharmacy	11 (6.4)

Marital status	Unmarried	96 (55.8)
	Married	63 (36.6)
	Divorced	13 (7.6)

Experience (years)	1–4	120 (69.8)
	5–10	34 (19.8)
	Above 10	18 (10.5)

Position	Owner	35 (20.3)
	Employee	137 (79.7)

Hours per day	<8	48 (27.9)
	≥8	124 (72.1)

Days per week	5	5 (2.9)
	6	100 (58.1)
	7	67 (39.0)
Mean working hours per week ± S.D.	59.15 ± 20.57	

Satisfaction	Yes	110 (64.0)
	No	62 (36.0)

Workload	Yes	138 (80.2)
	No	34 (19.8)

Extra allowance	Yes	45 (26.2)
	No	127 (73.8)

Chronic illness	Yes	9 (5.2)
	No	163 (94.8)

**Table 2:** Distribution of respondents' response on COVID-19 Peritraumatic Distress Index (CPDI).

Statements	Never	Occasionally	Sometimes	Often	Always
Compared to usual, I feel more nervous and anxious	21 (12.2)	28 (16.3)	83 (48.3)	24 (14.0)	16 (9.3)
I feel insecure and bought a lot of masks, medications, sanitizers, gloves, and/or other home supplies	20 (11.6)	19 (11.0)	46 (26.7)	41 (23.8)	46 (26.7)
I can't stop myself from imagining myself or my family being infected and feel terrified and anxious about it	31 (18.0)	33 (19.2)	38 (22.1)	39 (22.7)	31 (18.0)
I feel helpless no matter what I do	51 (29.7)	31 (18.0)	56 (32.6)	20 (11.6)	14 (8.1)
I feel sympathetic to COVID-19 patients and their families	14 (8.1)	23 (13.4)	39 (22.7)	24 (14.0)	72 (41.9)
I feel helpless and angry about people around me, governors, and media	30 (17.4)	21 (12.2)	38 (22.1)	35 (20.3)	48 (27.9)
I am losing faith in the people around me	49 (28.5)	26 (15.1)	60 (34.9)	23 (13.4)	14 (8.1)
I collect information about COVID-19 all day. Even if it's not necessary, I can't stop myself	26 (15.1)	26 (15.1)	54 (31.4)	26 (15.1)	40 (23.3)
I will believe the COVID-19 information from all sources without any evaluation	65 (37.8)	36 (20.9)	43 (25.0)	13 (7.6)	15 (8.7)
I would rather believe in negative news about COVID-19 and be skeptical about the good news	63 (36.6)	28 (16.3)	47 (27.3)	20 (11.6)	14 (8.1)
I am constantly sharing news about COVID-19 (mostly negative news)	60 (34.9)	38 (22.1)	50 (29.1)	13 (7.6)	11 (6.4)
I avoid watching COVID-19 news since I am too scared to do so	51 (29.7)	34 (19.8)	51 (29.7)	26 (15.1)	10 (5.8)
I am more irritable and have frequent conflicts with my family	72 (41.9)	30 (17.4)	47 (27.3)	19 (11.0)	4 (2.3)
I feel tired and sometimes even exhausted	29 (16.9)	43 (25.0)	72 (41.9)	18 (10.5)	10 (5.8)
When feelings anxious, my reactions are becoming sluggish	37 (21.5)	56 (32.6)	57 (33.1)	15 (8.7)	7 (4.1)
I find it hard to concentrate	31 (18.0)	43 (25.0)	64 (37.2)	24 (14.0)	10 (5.8)
I find it hard to make any decisions	31 (18.0)	45 (26.2)	68 (39.5)	22 (12.8)	6 (3.5)
During this COVID-19 period, I often feel dizzy or have back pain and chest distress	46 (26.7)	31 (18.0)	66 (38.4)	26 (15.1)	3 (1.7)
During this COVID-19 period, I often feel stomach pain, bloating, and other stomach discomforts	74 (43.0)	30 (17.4)	46 (26.7)	15 (8.7)	7 (4.1)
I feel uncomfortable when communicating with others	64 (37.2)	42 (24.4)	40 (23.3)	18 (10.5)	8 (4.7)
I talked with my family members very rarely	85 (49.4)	30 (17.4)	33 (19.2)	15 (8.7)	9 (5.2)
I have frequent awakening at night due to my dream about myself or my family being infected by COVID-19	65 (37.8)	27 (15.7)	55 (32.0)	22 (12.8)	3 (1.7)
I have changes in my eating habits	40 (23.3)	24 (14.0)	63 (36.6)	24 (14.0)	21 (12.2)
I have constipation or frequent urination	92 (53.5)	30 (17.4)	30 (17.4)	14 (8.1)	6 (3.5)

**Table 3:** Distribution of respondents on the severity of psychological distress.

Level of psychological distress	Frequency	Percentage
Mild to moderate (28–51)	38	22.1
Severe (52–100)	134	77.9

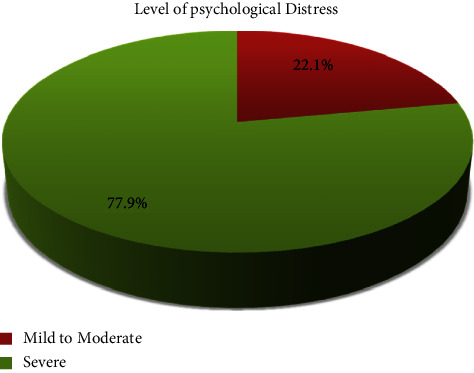		

**Table 4:** Association between level of psychological distress and sociodemographic and work-related characteristics of pharmacists.

Characteristics	Category	Mild/Moderate	Severe	Chi-square	*P* value
Sex	Male	16	54	0.040	0.841
	Female	22	80		

Age	Below 30	32	90	4.172	**0.041**
	Above and equal to 30	6	44		

Ethnicity	Brahmin	22	61	1.815	0.178
	Non-Brahmin	16	73

Religion	Hindu	32	109	0.165	0.685
	Non-Hindu	6	25		

Education	Diploma	27	62	7.283	**0.007**
	Bachelor & above	11	72		

Marital status	Unmarried	18	78	1.411	0.235
	Married/Divorced	20	56		

Position	Owner	9	26	0.335	0.563
	Employee	29	108		

Hours/day	<8	18	30	9.182	**0.002**
	8 and above	20	104		

Days/week	6 or less	26	79	1.116	0.291
	7	12	55		

Experience in years	3 or less	28	70	5.554	**0.018**
	<3	10	64		

Satisfaction	Yes	32	78	8.682	**0.003**
	No	6	56		

Work load	Yes	30	108	0.051	0.822
	No	8	26		

Allowance	Yes	10	35	0.001	0.981
	No	28	99		

Chronic illness	Yes	0	9	2.693	0.101
	No	38	125		

Statistical significance at *p* < 0.05.

**Table 5:** Bivariable and multivariable logistic regression examining the association between psychological distress and sociodemographic variables.

Study variable	Mild–Moderate	Severe	COR	*P* value	AOR	*P* value
			95% CI		95% CI	
Age (years)						
<30	32	90	1	0.047^*∗*^	1	0.799
≥30	06	44	2.607 (1.015–6.699)		0.854 (0.254–2.867)	

Education						
Diploma	27	62	1	0.008^*∗*^	1	0.002^*∗*^
Bachelor and above	11	72	2.850 (1.308–6.212)		4.489 (1.747–11.539)	

Hours						
<8 hours	18	30	1	0.003^*∗*^	1	0.00^*∗*^
≥8 or more hours	20	104	3.120 (1.466–6.640)		7.633 (2.729–21.352)	

Experience (years)						
≤3	28	70	1	0.021^*∗*^	1	0.038^*∗*^
>3	10	64	2.56 (1.153–5.684)		2.857 (1.060–7.702)	

Satisfaction to job						
Yes	32	78	1	0.005^*∗*^	1	0.00^*∗*^
No	06	56	3.829 (1.500–9.774)		11.524 (3.574–37.158)	

Statistical significance at *p* < 0.05^∗^; COR, crude odds ratio; AOR, adjusted odds ratio; CI, confidence interval; 1.00 reference group.

## Data Availability

The data used to support the findings of this study are included within the article.
